# The epitranscriptome in modulating spatiotemporal RNA translation in neuronal post-synaptic function

**DOI:** 10.3389/fncel.2015.00420

**Published:** 2015-10-31

**Authors:** Shobbir Hussain, Zafar I. Bashir

**Affiliations:** ^1^Department of Biology and Biochemistry, University of BathBath, UK; ^2^School of Physiology and Pharmacology, University of BristolBristol, UK

**Keywords:** epitranscriptome, epitranscriptomics, RNA methylation, synaptic plasticity, autism spectrum disorders, Intellectual Disability, NSun2, FMRP (FMR1)

## Abstract

The application of next-generation-sequencing based methods has recently allowed the sequence-specific occurrence of RNA modifications to be investigated in transcriptome-wide settings. This has led to the emergence of a new field of molecular genetics research termed “epitranscriptomics.” Investigations have shown that these modifications can exert control over protein synthesis via various mechanisms, and particularly when occurring on messenger RNAs, can be dynamically regulated. Here, we propose that RNA modifications may be a critical regulator over the spatiotemporal control of protein-synthesis in neurons, which is supported by our finding that the RNA methylase NSun2 colocalizes with the translational-repressor FMRP at neuronal dendrites. We also observe that NSun2 commonly methylates mRNAs which encode components of the postsynaptic proteome, and further find that NSun2 and FMRP likely share a common subset of mRNA targets which include those that are known to be translated at dendrites in an activity-dependent manner. We consider potential roles for RNA modifications in space- time- and activity-dependent regulation of protein synthesis in neuronal physiology, with a particular focus on synaptic plasticity modulation.

## Epitranscriptomics: A sleeping giant of fundamental molecular genetics research

The existence of covalent modifications that occur on DNA as well as those that occur on RNA molecules have been known about for well over half a century (He, 2010). Whilst only a very limited number of types of modification of DNA are known to exist, at least 130 types of RNA modification can occur, and indeed the frequency of modifications per base is also estimated to be much higher for various RNA biotypes, than for those occurring on the genome (Machnicka et al., 2013). But whereas molecular and biological investigations defining the form and function of the epigenome has excelled over the decades, research characterizing modifications occurring in the transcriptome had remained relatively dormant and this was largely due to technical limitations which hindered our ability to investigate the sequence-specific occurrence of these in any sufficient detail. A crucial event in the field was the demonstration that at least some of these modifications are reversible, thus suggesting that they may be dynamically controlled during the regulation of important biological processes (Jia et al., 2011). More recently, via various novel methodologies, researchers have managed to employ next-generation-sequencing based approaches to identify RNA modification sites in the transcriptome in sequence-specific contexts; this has included methyl-6-adenosine (m6A) modifications mainly found in mRNAs where it is enriched around stop codons (Dominissini et al., 2012; Meyer et al., 2012), methyl-5-cytosine (m5C) modifications present in tRNAs, mRNAs and a variety of ncRNAs (Squires et al., 2012; Hussain et al., 2013b; Khoddami and Cairns, 2013), pseudouridylations present in various ncRNAs and a number of mRNAs (Carlile et al., 2014; Schwartz et al., 2014), and very recently N^6^,2′-O-dimethyladenosine (m6Am) modifications present at the 5′ ends of some mRNAs (Linder et al., 2015). These key studies provided detailed transcriptomic RNA modification maps for the first time and have laid the foundations for further investigations to probe their mechanistic relevance in detail. Such studies may mark the dawn of a fledgling field of molecular genetics research coined “epitranscriptomics” (Saletore et al., 2012; Hussain et al., 2013c; Rinn and Ule, 2014).

Previous clues indicating the biological relevance of RNA modifications came from studies which investigated the function of enzymes that catalyze their deposition. This has been particularly noteworthy for m6A and m5C methylations which are currently the most well studied type of internal RNA modification. For m6A methylation, a complex which includes the METTL3 and METTL14 proteins, catalyzes the methyl transfer (Bokar et al., 1997; Liu et al., 2014) and is required for proper yeast meiosis (Clancy et al., 2002) and for plant and Drosophila development (Zhong et al., 2008; Hongay and Orr-Weaver, 2011). m5C modifications are catalyzed by methylases which in vertebrates include seven members of the NSun family (NSun1-NSun7) and the Dnmt2 enzyme. Of these RNA m5C-associated enzymes only Dnmt2 and NSun2, have been characterized in sufficient detail in higher eukaryotes. When Dnmt2 is deleted in flies, a reduced viability under stress conditions is observed (Schaefer et al., 2010). Mice lacking functional NSun2, display impaired differentiation in skin (Blanco et al., 2011), reduced fertility in males (Hussain et al., 2013a) and neurodevelopmental phenotypes (Blanco et al., 2014). Indeed, human patients lacking NSun2 are characterized by intellectual disability as well as other neurodevelopmental deficits (Abbasi-Moheb et al., 2012; Khan et al., 2012; Martinez et al., 2012; Fahiminiya et al., 2014; Komara et al., 2015). In addition, the expression of NSun2 is found to be enriched in the brain and dynamically regulated during early neural development in mice (Chi and Delgado-Olguín, 2013) further suggesting a particularly important role for this enzyme in neural function. For a more detailed overview of characterized RNA modification enzymes, the reader is referred to Blanco and Frye (2014) for an excellent review.

## The epitranscriptome in modulating protein synthesis

As indicated above, the epitranscriptome constitutes a huge number of diverse components, only four of which (m6A, m5C, pseudouridine, m6Am) have been characterized in the detail required to reliably investigate their biological roles. The modifications can occur on various types of non-coding as well as coding RNA, but so far investigations into function of modifications have overwhelmingly suggested multiple roles in the modulation of some aspect of protein-synthesis control (Tuorto et al., 2012; Hussain et al., 2013b,c; Blanco et al., 2014; Fu et al., 2014; Wang et al., 2014a, 2015), and some of the biological roles have been established to include regulation of circadian rhythms in hypothalamic suprachiasmatic nucleus in mouse brain (Fustin et al., 2013), as well as in the control of embryonic stem cell differentiation in studies utilizing cultured mouse embryonic stem cells (Wang et al., 2014b). Importantly, it also appears that these modifications, especially when occurring on mRNAs, can be dynamically regulated (Jia et al., 2011; Dominissini et al., 2012; Meyer et al., 2012; Hussain et al., 2013c; Carlile et al., 2014; Schwartz et al., 2014), and indicates the existence of enzymes that can both “write” and “erase” marks, in addition to specialized RNA-binding effector proteins that can “read” them (Fu et al., 2014). Such studies are especially welcome in our understanding of fundamental aspects of molecular genetics, as transcriptomic investigations have demonstrated that mRNA translation levels correlate quite poorly, and seemingly haphazardly, with cellular mRNA levels (Ingolia et al., 2009, 2011; Ingolia, 2014). A mechanistic role for dynamic m6A mRNA modifications and associated reader proteins in such discordance was recently demonstrated in somewhat spectacular fashion by the Chuan He laboratory (Wang et al., 2015). The authors showed that in addition to promoting interaction with the YTHFD2 RNA-binding protein, m6A modifications in mRNAs also promoted the interaction with a related reader, YTHFD1. Whereas binding of YTHFD2 accelerates mRNA decay, binding of YTHFD1 significantly speeds up the rate of translation in HeLa cells; and thus by enhancing binding of the YTHFD readers, translation occurs acutely from m6A-modified mRNAs during a time-limited window. RNA modifications thus appear to offer layers of control that might enable regulated protein synthesis to occur in a space- time- and signal-dependent manner, thus allowing for highly dynamic modes of mRNA translation to participate directly in complex cellular functions. A rather exciting possibility worth investigating is whether such mechanisms may also occur in neurons, where the acute and dynamic control of mRNA translation takes on specialized roles (Holt and Schuman, 2013). Indeed it has been shown that deletion of the FTO gene in mice, which is an m6A eraser, results in an impairment of dopamine receptor control of neuronal activity and behavioral responses (Hess et al., 2013), thus suggesting important roles for this type of RNA modification in the mammalian brain.

## Activity-dependent protein synthesis in neuronal dendrites: A brief history

External signal-dependent spatiotemporal protein synthesis is thought to play roles in crucial aspects of neuronal function with important implications for neurodevelopment and neurophysiology (Sutton and Schuman, 2006; Holt and Schuman, 2013; Jung et al., 2014). The first indications that protein synthesis may occur locally within neuronal dendrites came from the observations that mRNA (Bodian, 1965) as well polyribosomes (Steward and Levy, 1982) were located within them. Later studies were also able to demonstrate the presence of tRNAs, ribosomal proteins, and translation initiation and elongation factors within dendrites making it likely that they contained the full complement of factors for autonomous protein synthesis. This was confirmed by experiments showing that protein synthesis was able to occur in dendrites that had been severed from the cell body in culture (Torre and Steward, 1992) as well as in brain slices (Kang and Schuman, 1996), and importantly that this protein synthesis could be stimulated in response to neuronal activity (Weiler and Greenough, 1991, 1993; Feig and Lipton, 1993; Aakalu et al., 2001). Further, it was also shown that certain mRNAs could be transported to dendrites in response to synaptic activity, and it appears that the type of input-stimulus regulated by particular receptor mediated pathways can influence which mRNA subsets are transported to them (Tongiorgi et al., 1997; Tiruchinapalli et al., 2003; Antar et al., 2004), as well as which mRNAs are translated once they are there (Gong et al., 2006; Wang et al., 2009; Sarkar et al., 2010).

Although activity-dependent dendritic protein synthesis may regulate multiple aspects of neuronal physiology, much of our knowledge indicates a large role in the maintenance of long-lasting synaptic changes in dendrites such as Long-Term-Potentiation (LTP) as well Long-Term-Depression (LTD; Kang and Schuman, 1996; Huber et al., 2000; Raymond et al., 2000). Synaptic plasticity is associated with changes in postsynaptic receptor and ion channel density (Ju et al., 2004; Sutton et al., 2004, 2006; Raab-Graham et al., 2006) as well as dendritic spine morphology and changes in the numbers of synaptic connections (Engert and Bonhoeffer, 1999; Cruz-Martín et al., 2010). Studies that have attempted to investigate the identity of proteins that may be locally synthesized to mediate such processes have found that subsets of mRNAs located at dendrites commonly include those encoding synaptic signaling proteins, postsynaptic receptor subunits; ion channels; postsynaptic scaffolding proteins, cytoskeletal proteins, as well as translation factors (Tang and Schuman, 2002; Sutton and Schuman, 2006). However, mechanistic insights into the molecular processes occurring within neurons and specifically within post-synaptic compartments required to regulate and execute the induction of such post-synaptic changes, as well as the processes via which they are maintained in the long-term, are still very much the subject of active inquiry.

## Molecular mechanisms of dendritic protein synthesis and synaptic plasticity

It has been shown that some mRNAs can be packaged into ribonucleoprotein (RNP) complexes which can then be actively transported on microtubules along dendrites. Such RNPs are known to include the Fragile-X mental retardation protein, FMRP (discussed further below), the Zip-code Binding Protein 1, (ZBP1), and the cytoplasmic polyadenylation binding protein (CPEB). ZBP1 and CPEB have been shown to bind to 3′UTR elements in the beta-actin and MAP2 mRNAs respectively, in order to target their transport along dendrites (Ross et al., 1997; Eom et al., 2003; Huang et al., 2003). In fact, several sequence elements that are present in mRNA 3′UTRs that are targeted by various dendritic transport factors have been identified, and in some cases a single 3′UTR can contain several targeting elements, thus suggesting a complex mode of signaling that may allow for stimulus-specific mechanisms of transport (Tang and Schuman, 2002). The mechanisms via which specific stimuli can actually signal to the cell body for subsets of mRNAs to be transported and to modulate synaptic plasticity are however, currently poorly defined (Swanger and Bassell, 2013).

In the postsynaptic compartment, NMDA- and mGlu- receptor-mediated signaling regulate the activity of mTOR and ERK1/2 intracellular signaling pathways, which can activate general mechanisms of translation in dendrites by influencing the assembly of translation initiation factors; these are thought to be key mechanisms in the regulation of various forms of synaptic plasticity (Bramham and Wells, 2007). An additional important pathway involves activation of the BDNF receptor, TrkB, which can induce and maintain LTP in a protein-synthesis dependent manner (Figurov et al., 1996; Panja and Bramham, 2014). Indeed, it was recently shown that LTP consolidation in the dentate gyrus of rodents requires long-term BDNF-TrkB signaling *in vivo* (Panja et al., 2014). The study further demonstrated that long-term TrkB signaling leads to sustained activation of the MAP kinase-interacting kinase (MNK) signaling pathway which promotes both early-stage translation and also late-stage translation phases required for LTP consolidation. Thus, whereas our knowledge of the mechanisms that can regulate dendritic translation is improving, the molecular mechanisms via which specific stimuli can turn on the local translation of particular mRNAs required for synaptic modulation are not well-characterized, but they likely rely upon yet to be defined multiple distinct intracellular signaling pathways and molecular mechanisms (Swanger and Bassell, 2013).

## The translation-repressor FMRP in synaptic plasticity

FMRP deserves special consideration because it has been extensively studied and is associated with neurological disease, but because it also offers crucial insight into the molecular mechanisms via which stimulus-dependent translation may be turned on and off to regulate synaptic plasticity (Sidorov et al., 2013). FMRP is known to be a component of RNPs that transport at least some mRNAs to dendrites, and it may play an active role in this process. It has mainly been characterized, however as an mRNA binding protein which represses translation, and it likely participates in delivering mRNAs in their translationally-silent form to dendrites (Bagni and Greenough, 2005; Santoro et al., 2012). A study which used a high-stringency screen for mRNA interaction partners of FMRP identified 842 targets in the mouse brain and also showed that FMRP represses translation by halting the progression of ribosomes along the mRNA (Darnell et al., 2011). Upon the relevant synaptic stimulation, FMRP may dissociate from mRNAs, or lose its repression-capability via some other means, leading to translational re-activation (Santoro et al., 2012).

FMRP has been shown to be important for modulating synaptic plasticity, with a critical role in the modulation of LTD in the hippocampus and the cerebellum (Huber et al., 2002; Koekkoek et al., 2005). Important roles for FMRP in LTP modulation have also been described. It has been shown for example that activation of the BDNF-TrkB-MNK axis that regulates LTP via the early-stage translation activation phase is mechanistically mediated via triggering the release of the CYFIP1/FMRP repressor complex from the 5′cap of mRNAs (Panja et al., 2014). However, the exact mechanism via which FMRP selects its mRNA targets is not clearly defined; for example, Darnell et al. (2011) failed to identify any common sequence or structural motifs in their subset of identified mRNA targets. Similarly the molecular mechanisms by which FMRP de-repression of translation occurs is still being investigated; and mechanisms uncovered so far include the phosphorylation status of FMRP (Ceman et al., 2003), the regulation of its interaction with a neuronal ncRNA, BC1 (Zalfa et al., 2003), as well as interactions with microRNAs (Ishizuka et al., 2002; Jin et al., 2004).

## Potential roles for RNA modifications in the modulation of synaptic function

In this section we focus on the major aims of this perspective article to introduce the potential roles of RNA modifications in local protein synthesis-dependent post-synaptic signaling, and to highlight what advantages such mechanisms may offer compared to other already known modes of regulation.

### Potential mechanisms and advantages offered by RNA modifications

Along with RBP's, miRNAs are also currently thought to be important regulators of translation in synaptic function (Schratt, 2009), although practically, their low abundance in dendrites and generally short half-life may put limitations on their functional applicability. RNA modifications may overcome some of these limitations, and may further allow for highly dynamic modes of translation control via multiple mechanisms by for example regulating methylation/demethylation events, of not only mRNAs, but also tRNAs and rRNAs [the reader is referred to Wang and He (2014) for an excellent recent perspective]. tRNAs in particular, but also rRNAs, are strewn with various modifications which are potentially reversible (Chan et al., 2010; Fu et al., 2010; Saikia et al., 2010). It is rather tempting to speculate that such dynamic modification events on tRNAs and rRNAs may be a mechanism to turn general dendritic/synaptic translation on and off i.e. by modulating interactions with other important components of the translation machinery that are present in dendrites or certain dendritic locations. It may further be noteworthy in this regard that the methylation activity of RNA methylases such as NSun2, whose targets include tRNAs, can also be modulated by phosphorylation status (Sakita-Suto et al., 2007), given that phosphorylation cascades are thought to be important players in post-synaptic signaling pathways that regulate translation.

Given the huge number of different types of RNA modifications possible, highly complex degrees of regulation might also be enabled with regards to regulated translation of specific mRNAs. It is worth considering for example whether an “epitranscriptome code” exists that may regulate complex aspects of mRNA translation in neurons, similarly to how the histone code may regulate complex aspects of regulated gene transcription in important biological processes. Such a code may for example enable the programming of dendrites of a neuron in a precisely specified manner, and may even allow different locations of the dendritic tree to have contrasting responses to the same extrinsic cue. For example, if the presence of a particular mRNA was important for post synaptic signaling in two different locations of the post-synaptic compartment of a neuron, then by applying a distinct pattern (or code) of modifications to the mRNA, the two locations might be able to respond differentially even though they are exposed to the same pre-synaptic signal. The mechanistic aspects of such regulation may vary, but could involve the ability of different modifications to affect recruitment to specific dendritic/synaptic sites, the ability to differentially recruit translation initiators/inhibitors, or the signaling of long-term stabilization or decay of transcripts. Although, such ideas are intriguing, especially given the observation of different patterns of synaptic plasticity induction even in spines along a single dendrite (Harnett et al., 2012), it will first be important to understand mechanistically how different modifications regulate aspects of RNA translation, and secondly whether indeed these mechanisms occur in neurons and particularly in dendrites/spines.

### Potential roles for the intellectual disability gene NSun2?

The NSun2 RNA methylase which catalyzes the formation of m5C is particularly interesting with regards to neural function, given its roles in human intellectual disability syndromes (Abbasi-Moheb et al., 2012; Khan et al., 2012; Martinez et al., 2012; Fahiminiya et al., 2014; Komara et al., 2015), and in some patients autistic features as well epileptic seizures have been reported (Martinez et al., 2012). In addition, in Drosophila and mouse models of NSun2-deficiency, deficits in memory and learning are also apparent (Abbasi-Moheb et al., 2012; Blanco et al., 2014). Intriguingly, it has been shown that lack of NSun2 in mice leads to fragmentation of tRNAs which may trigger stress responses and apoptosis in the brain (Blanco et al., 2014) and also a reduction in global translation rates (Tuorto et al., 2012), although the degree to which such a mechanism might contribute to the observed intellectual disability phenotypes remains unknown. It has also been shown that lack of NSun2 mediated methylation of the Vault ncRNA leads to mis-regulation of non-canonical microRNA production pathways which can modulate the cellular levels of the transmembrane AMPA receptor regulatory protein CACNG8 (Hussain et al., 2013b) known to regulate both trafficking and channel gating of AMPA receptors; however again, it remains unclear what roles such mechanisms play in NSun2-deficiency neurological phenotypes. Nonetheless, the roles of RNA modifications such as those mediated by NSun2 in brain function clearly represents an exciting area of future research likely to have significant impact on our understanding of neurological disease (Satterlee et al., 2014).

Transcriptomic investigations have indeed demonstrated that the vast majority of NSun2 methylation targets are those involved in some aspect of translation and include tRNAs, 5S rRNA, RPPH1 ncRNA, Vault ncRNA, and mRNA coding sequence (Squires et al., 2012; Hussain et al., 2013b; Khoddami and Cairns, 2013). As discussed above we are only just starting to elucidate some of the mechanistic roles of such modifications, and the early studies have focussed on tRNA methylation and methylation of the Vault ncRNA (Tuorto et al., 2012; Hussain et al., 2013b; Blanco et al., 2014). Further indications that NSun2 may be involved in intricate mechanisms of translation regulation are suggested by studies in testis which demonstrated that the enzyme was a component of the chromatoid body (CB) found in the cytoplasm of round spermatids (Hussain et al., 2013a). This was especially interesting as the CB is known to be a center for regulating the temporal control of mRNA translation in a space-restricted manner during the regulation of spermatogenesis (Kotaja and Sassone-Corsi, 2007; Nguyen Chi et al., 2009). Taken together with the findings that NSun2 is important for neurodevelopment, here we speculate that NSun2 may similarly play a role in spatiotemporal protein synthesis in neurons. Indeed, given that activity-dependent post-synaptic modulation is a key aspect of neuronal function known to rely heavily on spatiotemporal regulation of protein synthesis, we hypothesize that NSun2-mediated methylation may act as a point of control in these pathways.

### Some empirical support

**Figure 1:**
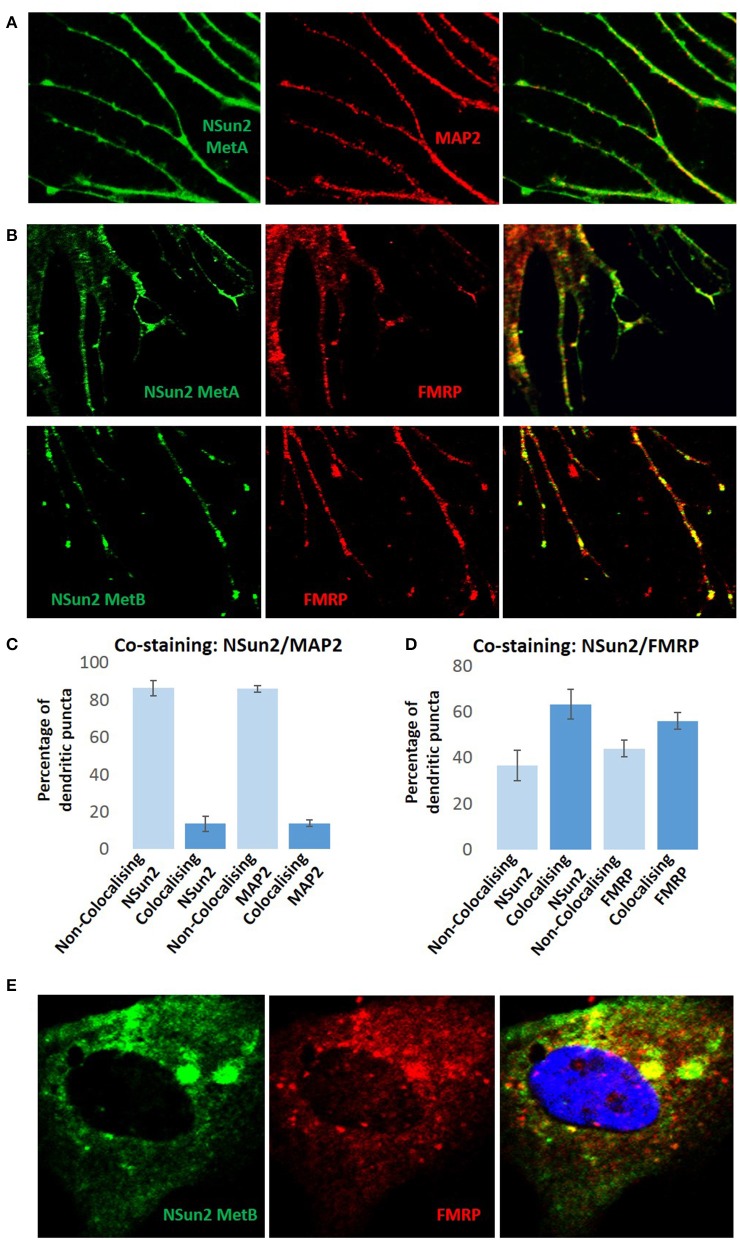
**NSun2 localizes to neuronal dendrites**. The human fetal brain-derived neural stem cell line CB660 (Sun et al., 2008) was maintained in RHB-A medium (Stem Cell Sciences, UK) supplemented with FGF2 and EGF on laminin-coated culture flasks. In order to induce differentiation, the maintenance medium was replaced with neurobasal medium supplemented with N2, B27, and FGF2. FGF2 was then removed from the medium and BDNF added in a step-wise manner to induce neuronal differentiation as previously described (Sun et al., 2008). Immunofluorescent-staining procedures and imaging using a confocal microscope were performed as described previously (Hussain et al., 2013a). **(A)** In differentiated neurons, NSun2 localizes to dendrites which were specified using the dendritic marker MAP2 (Abcam, HM-2). **(B)** We also find partial colocalization with FMRP (Thermo Scientific, 4G9) at dendrites. For the immuno-detection of NSun2, two previously characterized NSun2 antibodies were used (NSun2 MetA and NSun2 MetB; Frye and Watt, 2006). The extent of NSun2/MAP2 colocalization in **(C)** and NSun2/FMRP colocalization in **(D)** was assessed by counting the number of colocalizing/non-colocalizing synaptic puncta in dendrites. Quantifications represented are the average from three independent immunostaining experiments, and error bars display standard deviation from the mean. **(E)** NSun2 also displays more modest levels of colocalization with FMRP in cell bodies of neurons. DAPI was used as a counterstain for nuclei (right panel, blue).

**Figure 2:**
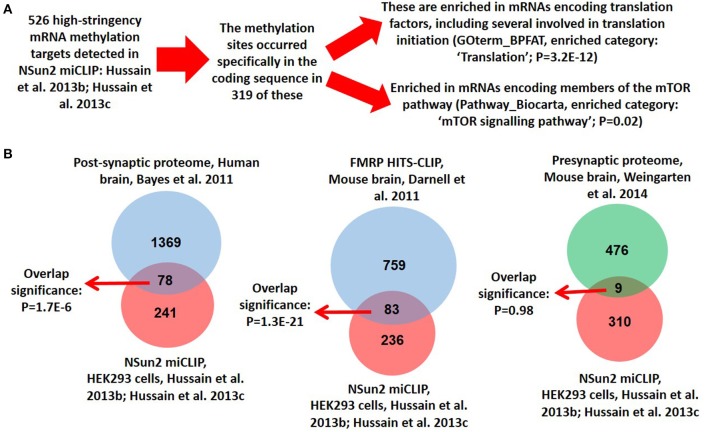
**Analysis of NSun2 mRNA methylation targets**. **(A)** Methylation-individual-nucleotide-resolution-crosslinking-immunoprecipitation (miCLIP) was previously used to determine the transcriptomic RNA methylation targets of NSun2 in HEK293 cells (Hussain et al., 2013b). Most NSun2 methylation sites occurred within the coding sequence of mRNAs, and 319 of these were identified (Hussain et al., 2013b,c); these are found to be enriched in mRNAs encoding translation factors as well members of the mTOR signaling pathway. Gene Ontology analysis and calculation of *P*-values for enrichment was performed by the DAVID Bioinformatics Resource. Full gene lists available in Supplementary Table 1. **(B)** The 319 NSun2 mRNA methylation targets from HEK293 cells were compared with the 1461 members of the postsynaptic proteome from the human neocortex (Bayés et al., 2011), the 842 FMRP mRNA binding targets from the mouse brain (Darnell et al., 2011), and the 485 members of the pre-synaptic proteome from the mouse brain (Weingarten et al., 2014). Full gene lists available in Supplementary Table 1. Considering the shared expression of >90% of mRNAs between HEK293 cells and brain (Su et al., 2004), and using a conservative approximation of 10,000 protein coding genes expressed in each (Ramsköld et al., 2009), a hypergeometric test was used to calculate the significance of the degree of overlap observed between the targets.

In support of such a perspective, here we show that NSun2 localizes to dendrites of neurons in culture (Figure 1A), where it was found to partially colocalize with FMRP (Figure 1B). Quantification of dendritic puncta revealed that whereas NSun2 displayed limited levels of colocalization with MAP2 puncta, a much more significant degree of colocalization was observed with FMRP puncta suggesting that the observed partial colocalization with this protein was not co-incidental (Figures 1C,D). NSun2 was also observed in cell bodies of neurons, where a much more moderate level of colocalization with FMRP puncta was observed (Figure 1E) compared to in dendrites, and also in axons where there was no appreciable colocalization with FMRP (data not shown). By further analysing our previously published transcriptomic data (Hussain et al., 2013b,c), we further find that the mRNA coding sequence methylation targets of NSun2 in HEK293 cells are enriched in those encoding translation factors as well as those of the mTOR pathway (Figure 2A and Supplementary Table 1). Enrichment of mTOR mRNAs as NSun2-methylation targets is particularly interesting as it is known that these can be translated in dendrites in an activity-dependent manner (Khan et al., 2001; Li et al., 2010); such targets were enriched for members of the mTORC1 pathway in particular and included mTOR, TCS2, and RPS6 mRNAs. We also find a very high degree of overlap between NSun2-mRNA methylation targets identified in HEK293 cells with members of the post-synaptic proteome from the human neocortex (Bayés et al., 2011), and also with FMRP-mRNA binding targets identified by HITS-CLIP from the mouse brain (Darnell et al., 2011; Figure 2B and Supplementary Table 1). Although HEK293 cells resemble neuronal lineage cells (Shaw et al., 2002; Thomas and Smart, 2004), this latter finding though somewhat encouraging, should be viewed with some caution given the distinct systems analyzed in the different studies. Though incidentally, we do not observe any significant overlap of components of the presynaptic proteome in the mouse brain (Weingarten et al., 2014) with NSun2-methylation targets (Figure 2B and Supplementary Table 1).

That NSun2 may function in FMRP-regulated post-synaptic pathways such as synaptic plasticity modulation is an intriguing prospect since NSun2 displays highest expression in the hippocampus of the mouse brain (Allen brain atlas) and especially given the shared phenotypes of human patients deficient in these genes (Komara et al., 2015 for an overview of NSun2-deficiency syndromes). However, it will first be important to determine the targets of NSun2 from the relevant brain tissue, and the nature of the neurophysiological disturbance in NSun2-deficient brains, before any mechanistic insights, such as a potential link to the FMRP pathway, can be adequately gleaned and pursued.

## Future directions

Advances in epitranscriptomics clearly have the potential to offer new perspectives of important physiological processes, particularly with regards to aspects of neuronal function where features of RNA function appear to take on specialized roles. A careful study into the neurological functions of the NSun2 RNA methylase may prove a seminal example. The first key questions that need to be addressed is what the transcriptomic RNA methylation targets in the brain are: tRNAs? rRNA? Vault RNA? mRNAs? Other neuron-specific RNAs not yet detected? What link, if any, can be made to characterized pathways such as the FMRP-pathway? And then what are the disturbances of translation at the transcriptomic level? Is there for example a global reduction of translation? Or is there a correlation specifically to direct mRNA methylation targets, or those targeted by Vault RNA-derived microRNAs? Powerful and accurate transcriptomic approaches such as miCLIP (Hussain et al., 2013b) and nucleotide-resolution ribosomal profiling (Ingolia et al., 2009), should have the capability to answer such questions in a comprehensive and reliable manner. However, such studies alone are unlikely to provide the necessary knowledge required to gain a reliable footing on the nature of the neurological disturbance in NSun2-defecient brains. Given the localization of NSun2 to dendrites, and the fact that it is a translation regulator, a role in the regulation of some aspect of translation-dependent postsynaptic function seems likely. For this purpose, electrophysiological experiments would help to uncover the nature of the physiological defect: is there a mis-regulation of known forms of protein synthesis-dependent LTP or LTD for example? Or some other aspect of local protein-synthesis-mediated post-synaptic signaling or synaptic formation?

Following such investigations we will be in a position to design experiments to ascertain the mechanistic roles of RNA methylation in post-synaptic function and disease pathogenesis. A better understanding of the molecular pathways that regulate synaptic plasticity may indeed have important implications for understanding the molecular pathogenesis of more common neurodevelopmental disorders such as autism spectrum disorders (ASDs), where the disruption of RNA translation-dependent synaptic plasticity pathways has already been suggested and indicated by empirical evidence (Bear et al., 2008; Darnell and Klann, 2013; Gkogkas et al., 2013; Santini et al., 2013). Finally, although much of this article has focussed on m5C modifications and NSun2, we should remember that we are only at the beginning of the epitranscriptomics journey. Several other RNA-modifying enzymes exist, and determining the roles of the 100+ types of RNA modifications we know about seems a daunting task. But they likely provide the arena of complexity we have for long felt must exist in our RNA world (He, 2010), and accordingly offer the opportunities to better understand some important aspects of neuronal function.

### Conflict of interest statement

The authors declare that the research was conducted in the absence of any commercial or financial relationships that could be construed as a potential conflict of interest.

## Supplementary material

Supplementary Table 1 **miCLIP-generated NSun2 mRNA-target lists, including lists generated following DAVID Bioinformatics analysis, and comparison with post-synaptic proteome, presynaptic proteome, and FMRP targets**.
